# Higher blood pressure in elderly hypertensive females, with increased arterial stiffness and blood pressure in females with the Fibrillin-1 2/3 genotype

**DOI:** 10.1186/s12872-020-01454-9

**Published:** 2020-04-17

**Authors:** Ida Åström Malm, Urban Alehagen, Peter Blomstrand, Ulf Dahlström, Rachel De Basso

**Affiliations:** 1 School of Health and Welfare, Jönköping University, Jönköping, Sweden; 2 Department of Medical and Health Sciences, Linköping University, Linköping, Sweden; 3 Department of Clinical Physiology, County Hospital Ryhov, Jönköping, Sweden; 4 Department of Cardiology Linköping University, Linköping, Sweden

**Keywords:** Arterial stiffness, Elderly, Female, Fibrillin-1, Hypertension

## Abstract

**Background:**

Elderly patients have a relatively high cardiovascular risk due to increased arterial stiffness, elevated blood pressure and decreased amounts of elastin in the arteries. The composition of the media layer in the arterial wall, comprising elastin, collagen, smooth muscle cells, proteoglycans, fibronectin and fibrillin-1, influences its mechanical properties. Mutations in the fibrillin-1 gene leads to increased aortic stiffness, elevated pulse pressure and aortic root dilatation. This study investigates whether there is a sex difference among hypertensive elderly patients regarding blood pressure, arterial stiffness and fibrillin-1 genotypes.

**Methods:**

A total of 315 hypertensive subjects (systolic blood pressure > 140 mmHg) were included in this study (155 men and 160 women aged 71–88 years). Aortic pulse wave velocity and augmentation index were determined using SphygmoCor, and brachial blood pressure was measured using an oscillometric technique. Fibrillin-1 was genotyped by polymerase chain reaction and with a capillary electrophoresis system.

**Results:**

Females showed a significantly higher peripheral mean arterial pressure (females; 107.20 mmHg, males 101.6 mmHg, *p* = 0.008), central mean arterial pressure (females; 107.2 mmHg, males 101.6 mmHg *p* = 0.008), central systolic blood pressure (females; 148.1 mmHg, males 139.2 mmHg, *p* <  0.001) and central pulse pressure (females; 68.9 mmHg, males 61.6 mmHg, *p* = 0.035) than males. Females with the Fibrillin-1 2/3 genotype showed a significantly higher augmentation index (FBN1 2/3; 39.9%, FBN1 2/2 35.0%, FBN1 2/4 35.8, *p* = 0.029) and systolic blood pressure (FBN1 2/3; 174.6 mmHg, FBN1 2/2168.9 mmHg, FBN1 2/4169.9 mmHg, *p* = 0.025) than females with the 2/2 and 2/4 genotypes.

**Conclusion:**

The findings of this study may indicate that hypertensive elderly females, especially elderly females with Fibrillin-1 2/3, have increased systolic blood pressure and arterial stiffness.

## Background

It is still unclear whether arterial wall stiffness is a cause or a consequence of high blood pressure but the two conditions are close associated to each other [1]. Increased resistance in the small arteries enhance mean blood pressure which increase arterial wall stiffness and pulse wave velocity in the large elastic arteries. This raises central blood pressure further which might lead to organ injuries and damage to the small resistance vessels. Hypertension is a common disease among elderly individuals and is an increasing public health concern [2]. Thus, it is important to identify new potential biomarkers to detect preclinical disease and prevent hypertension. Arterial wall stiffness is an independent predictor of cardiovascular morbidity and mortality, increasing with age and is observed among elderly [3, 4]. An increased arterial stiffness and pulse wave velocity (PWV) cause an early return of the reflected pulse wave in late systole, which increases the central pulse pressure, myocardial oxygen demand and left ventricular workload. Some clinical manifestations of decreased arterial distensibility are hypertension and elevated pulse pressure (PP) [5].

Similar to arterial stiffness, systolic blood pressure (SBP) increases with age [6]. Elderly patients have a higher cardiovascular risk, through increased arterial wall stiffness, elevated blood pressure and decreased levels of elastin in their large elastic arteries [7–9]. Males have a higher SBP than females throughout most of their lives; however, after 65 years of age, the difference between sexes seems to disappear, and after the age of 70, females may have a higher prevalence of hypertension [10–12]. Thus, it is of interest to study the differences in the mechanical properties of arteries between the sexes among elderly patients with high SBP.

Mutations in the fibrillin-1 (FBN1) gene is associated with increased aortic stiffness, elevated PP and aortic root dilatation [13, 14]. Several studies have found an association between the FBN1 2/3 genotype and arterial stiffness in males. Medley et al. (2002) and Powell et al. (1996) reported a relationship between the 2/3 genotype and an increased PP [15, 16], and in a later study an association between the 2/3 genotype and an increased heart rate (HR), blood pressure and abdominal aortic stiffness in apparently healthy first-degree male relatives of patients with abdominal aortic aneurysms (AAAs) was reported [17]. In a study of both sexes, no associations between the FBN1 2/3 genotype and increased aortic PWV were observed [18]. The aim of this study was to investigate whether there is a sex difference regarding blood pressure, arterial stiffness and the FBN1 genotypes in an elderly hypertensive population.

## Methods

### Study population

The study population was recruited from an ongoing longitudinal study of elderly community-living people from a rural municipality of southeast Sweden. Inhabitants aged 65–82 years were invited to join the original study between 1998 and 2001. A total of 1130 individuals were invited, and 876 of these individuals agreed to participate in the study [19]. In a follow-up study, from 2003 to 2005, a cross-sectional study with the same population was performed. Before the follow-up study started, 123 study subjects had died. Of the remaining 753 subjects, 452 subjects agreed to participate in the current study. Twenty-three subjects were excluded because of irregular HR with variable PP in the arteries, hepatitis infection or difficulties obtaining blood samples. Among the remaining 429 subjects, the abdominal aorta was examined using ultrasound. After the ultrasound examination, an additional 23 subjects were excluded due to low-quality ultrasonic measurements [20]. In this study, we investigated subjects with SBP > 140 mmHg, and the final study population was 315 subjects (155 men and 160 women) within the age group of 71–88 years [20, 21].

The subjects were instructed not to use tobacco or consume coffee or tea for at least 4 h prior to their examinations. In addition, the participants had a physical examination and were asked about cardiovascular risk factors, cardiovascular diseases and medications. Subjects with previously diagnosed hypertension or with an SBP ≥ 140 mmHg were defined as hypertensive. Diabetes mellitus was defined as a previous diagnosis of diabetes mellitus or with ≥7 mmol l^− 1^ fasting plasma glucose. Ischemic heart disease was defined as a previous diagnosis of ischemic heart disease confirmed by exercise ECG or coronary angiography or electrocardiographically (ECG) verified myocardial infarction. Subjects who reported that they smoked were classified as smokers. Height and weight were measured and used for calculation of body mass index (BMI). All subjects in the study gave their written informed consent [20, 21]. The study was approved by the Regional Ethical Review board in Linköping, Sweden, and was conducted in accordance with the principles stated in the Declaration of Helsinki.

### Measurements

All measurements were performed under standardized conditions. Brachial blood pressure was measured after at least 15 min of rest when the subject was in the supine position using an oscillometric technique (Dinamap model PRO 200 Monitor, Critikon, Tampa, FL, USA). Systolic, diastolic and mean arterial pressures were measured [20, 21].

The ankle brachial pressure index (ABPI) was calculated using the SBP measured from the dorsalis pedis and the posterior tibial arteries recorded by a hand-held 8 MHz Doppler probe and a 12-cm standard cuff divided by the brachial systolic pressures measured in the right and left arm [20, 21].

Radial artery pulse waves were obtained noninvasively using the SphygmoCor system (version 7.0, Model MM3, AtCor Medical, Sydney, Australia). The SphygmoCor system was equipped with a Millar pressure tonometer. For PWV, the central pressure waveform was derived using a generalized transfer function, calculated from an 11-s recording of the radial artery pressure. For calibration of the pressure wave, brachial systolic and diastolic blood pressure were used. Blood pressure was measured before the pulse wave recordings. Registrations of pulse waves were repeated at least three times, and average data from the three most successful registrations were used for analysis. Only good-quality recordings were used for pulse wave analysis (PWA). The augmentation index (AIx) and pressure augmentation (AG) were calculated from the central pressure waveform. AIx is the pressure augmentation expressed as the percentage of the central pulse pressure (cPP): AIx = (AG/PP) × 100 [20, 21].

### Blood samples

Blood samples were taken after overnight fasting and collected in prechilled plastic Vacutainer tubes (Terumo EDTA K-3). Plasma was prepared by centrifugation at 3000 g for 10 min at 4 °C. Blood and plasma were stored at − 70 °C pending analysis. Plasma levels of fasting glucose, low-density lipoprotein (LDL) and high-density lipoprotein (HDL) were measured [20, 21].

### FBN1 genotyping

FBN1 genotyping was performed in 2009, and the investigators performing these assays were blinded to the results of the vascular testing. DNA was prepared for polymerase chain reaction (PCR) by typing the variable tandem nucleotide repeat (VNTR) (TAAAA)_n_ in intron 28 of the FBN1 gene on chromosome 15 using the forward primer 5′ 6FAM – CAG AGT ACA TAG AGT GTT TTA GGG AGA − 3′ and the reverse primer 5′- GTT TCT TCC TGG CTA CCA TCC AAC TCC c-3′. PCR was performed in a volume of 12 μl with predenaturation at 95 °C for 9 min; 35 cycles of denaturation for 30 s, annealing at 65 °C for 30 s (35 cycles), and extension at 72 °C for 30 s (35 cycles); and a final extension at 72 °C. A 1-μl portion of the PCR product was diluted with 9 μl of highly deionized formamide (GENESCAN™ 500ROX™ Size Standard) for electrokinetic injection on the capillary electrophoresis system. The DNA fragments were labeled with the ROX™ fluorophore. The labeling results in a single peak under denaturing conditions were obtained, and the alleles were then identified by the number of base pairs (bp) comparable to each peak (2/2: 162 bp, 162 bp; 2/3: 157 bp, 162 bp; and 2/4: 152 bp, 162 bp) [20–22].

### Statistics

All data are presented as the means±SDs. All continuous variables were checked for normality distribution, a log-transformation was used in continuous variables with a non-normality distribution. Student’s t-test and Mann–Whitney U-test were used to calculate differences between sexes. One-way ANOVA with post hoc testing was used to compare the differences between the genotypes. Variance analysis was used to adjust the dependent variables (peripheral mean arterial pressure (pMAP), central mean arterial pressure (cMAP), central SBP (cSBP) and cPP for age, body surface area (BSA) and HR). *p*-values < 0.05 were considered statistically significant. The statistical analyses were performed with IBM SPSS 25.0.

## Results

### Demographic data

A total of 315 hypertensive subjects (155 males/160 females; mean age, 78 ± 3.4 years) were analyzed, and demographic data according to sex are presented in Table 1. There were no significant differences between sexes regarding age, BMI, CRP, glucose, diabetes, hyperlipidemia or angina pectoris. Male subjects had a higher frequency of smokers (*p* = 0.005) and higher creatinine (*p* <  0.001) than female subjects, whereas females had higher HDL (*p* <  0.001) and LDL (*p* = 0.048) than males. Males were more affected by myocardial infarction (*p* = 0.021) than females. However, regarding heredity for cardiovascular disease, females showed increased family history of CV (*p* = 0.026) compared to males. Furthermore, there were no significant differences between males and females regarding treatments with statins, beta receptor blockers and ACE inhibitors. However, females were more commonly treated with diuretics than males (*p* = 0.045).

**Table 1:** Characteristics according to sex

	All *n* = 315	Male *n* = 155	Female *n* = 160	*p*-value
Age	78 ± 3.44	78 ± 3.18	78.5 ± 3.68	0.32
BMI	26.51 ± 3.90	26.16 ± 3.01	26.87 ± 4.59	0.14
Weight	74.01 ± 12.63	78.99 ± 9.20	69.18 ± 13.13	< 0.001
Height	166.95 ± 9.28	174.77 ± 6.91	160,35 ± 5.90	< 0.001
CRP (mg/l)^a^	5.1 (11.13)	5.25 (12)	45 (18.9)	0.82
Glucose (mmol/l)	5.81 ± 2.29	5.86 ± 2.10	5.75 ± 2.46	0.68
HDL (mmol/l)	1.30 ± 0.33	1.19 ± 0.26	1.4 ± 0.36	< 0.001
LDL (mmol/l)	3.18 ± 1.0	3.07 ± 0.89	3.29 ± 1.08	0.048
Creatinine (μmol/l)	79.51 ± 24.75	88.07 ± 24.88	71.23 ± 21.67	< 0.001
GFR (ml/min/1.73m^2^)	72.0 ± 22.1	74.5 ± 19.5	69.7 ± 24.2	0.057
History n (%)				
Smoking *	27 (8.6)	22 (14.2)	5 (3.1)	0.005
Diabetes*^b^	57 (18.1)	28 (18.1)	29 (18.1)	0.46
Hyperlipidemia *	73 (23.2)	32 (20.6)	41 (25.6)	0.51
Angina pectoris *	56 (17.8)	28 (18.1)	28 (18.1)	0.44
Myocardial infarction*^3^	27 (8.6)	19 (12.3)	8 (5.0)	0.021
Family history of CV *^4^	65 (20.6)	24 (15.5)	41 (25.6)	0.026
Medications, n (%)				
Statin treatment *	69 (21.9)	32 (20.6)	37 (23.1)	0.40
Beta receptor blockers*	114 (34.9)	54 (34.8)	60 (37.5)	0.63
ACE inhibitors*	62 (17.5)	27 (17.4)	35 (21.9)	0.15
Calcium channel blockers*	51 (16.2)	27 (17.4)	24 (15)	0.65
Diuretics*	115 (36.5)	48 (31)	67 (41.9)	0.045

Data are the means ±SD, frequencies and percentages* or as median and (range) ^a^. HDL; high-density lipoprotein, LDL; low-density lipoprotein. ^b^Diagnosis alt. > 7 mmol. ^3^ECG-verified. ^4^Family history of CV; Family history of cardiovascular disease.

### Blood pressure and arterial stiffness

Data on pressure and arterial stiffness divided by sex are presented in Table 2. When the SphygmoCor technique was used, the data showed that females had significantly higher pMAP (*p* = 0.008) and cMAP (*p* = 0.008) than males. These sex differences were also observed for cSBP (*p* <  0.001) and cPP (*p* = 0.035). After adjustments for age, HR and BSA, these differences were strengthened (pMAP *p* = 0.005; cMAP *p* = 0.005; cSBP *p* = 0.001; cPP *p* <  0.001). Differences regarding AIx (*p* <  0.001), AG (*p* <  0.001) and transit time (TR) (*p* <  0.001) arterial stiffness were observed between sexes.

**Table 2:** Pressure and arterial stiffness related to sex differences

	Male *n* = 155	Female *n* = 160	*p*-value
pMAP (mmHg)	101.55 ± 10.58	107.19 ± 13.50	0.008
cSBP (mmHg)	139.22 ± 16.86	148.09 ± 20.06	< 0.001
cDBP (mmHg)	77.65 ± 9.52	79.16 ± 11.22	0.33
cPP (mmHg)	61.56 ± 15.22	68.93 ± 17.03	0.035
cMAP (mmHg)	101.56 ± 10. 58	107.19 ± 13.50	0.008
AIx (%)	32.52 ± 8.73	36.04 ± 9.22	< 0.001
AI hr75 (%)	26.18 ± 7. 39	32.73 ± 6.91	< 0.001
HR (beat/min)	62.0 ± 11.38	68.06 ± 12.01	< 0.001
TR time (s)	133.26 ± 10.29	127.03 ± 9.29	< 0.001
AG (mmHg)	20.45 ± 9.37	25.50 ± 10.35	< 0.001
SBP right (mmHg)	162.13 ± 19.14	169.57 ± 23.51	0.004
SBP left (mmHg)	158.19 ± 16.76	165.18 ± 20.40	0.002
DBP right (mmHg)	80.31 ± 9.58	80.38 ± 11.27	0.91
DBP left (mmHg)	79.38 ± 9.36	79.10 ± 11.06	0.67
ABPI right (mmHg)	1.067 ± 0.25	1.03 ± 0.19	0.57
ABPI left (mmHg)	1.05 ± 0.22	1.02 ± 0.18	0.42

All data are presented as the means ± SDs. *pMAP* Peripheral mean arterial pressure, *cSBP* Central systolic blood pressure, *cDBP* Central diastolic blood pressure, *cPP* Central pulse pressure, *cMAP* Central mean arterial pressure, *AIx* Augmentation index, *AI hr75* Augmentation index heartrate 75, *HR* Heartrate, *TR time* Transistor time, *AG* Augmentation. Pressure: *SBP* Systolic blood pressure, *DBP* Diastolic blood pressure, *ABPI* Ankle brachial pressure index.

Using the oscillometric technique, the SBP was higher among female subjects than among male subjects (*p* = 0.003), and this difference was maintained after adjustments for age, HR and BSA (*p* = 0.006). Regarding diastolic blood pressure, no significant difference was found between sexes.

### FBN1

Four alleles and eight combinations of FBN1 genotypes were identified (1/2, 1/4, 2/2, 2/3, 2/4, 3/3, 3/4, and 4/4), and the three most common genotypes (2/2, 2/3 and 2/4) accounted for 79.4% of the population (males 75.4%, females 83.1%). Subsequent analysis was restricted to individuals with the three most common FBN1 genotypes 2/2, 2/3 and 2/4 and were divided by sex. Data on pressure and arterial stiffness according to FBN1 genotype and sex are presented in Tables 3 and 4. The frequencies of the genotypes were 2/2: 60.7% (males), 61.7% (females); 2/3: 14.5% (males), 13.5% (females); and 2/4: 34.2% (males), 24.8% (females).

**Table 3:** Pressure and arterial stiffness according to FBN1 genotype in males

	2/2 genotype	2/3 genotype	2/4 genotype	
	*n* = 71	*n* = 14	*n* = 40	*p*-value
pMAP (mmHg)	102.4 ± 10.5	102.6 ± 15.5	100.3 ± 10.0	0.23
cSBP (mmHg)	139.6 ± 18	137.1 ± 23.3	138.3 ± 15.2	0.83
cDBP (mmHg)	78.2 ± 9.1	79.5 ± 13	77.1 ± 9.4	0.29
cPP (mmHg)	61.4 ± 16.7	57.6 ± 16.3	61.2 ± 12.8	0.67
cMAP (mmHg)	102.4 ± 10.5	102.6 ± 15.9	100.3 ± 9.9	0.60
AIx (%)	30.22 ± 9.47	34.11 ± 9.28	33.70 ± 7.71	0.052
AI hr75 (%)	25.8 ± 8.0	28.3 ± 6.6	26.8 ± 6.6	0.52
HR (beats/min)	66.0 ± 11.6	62.8 ± 10.8	60.5 ± 9.8	0.036
TR time (s)	132.9 ± 9.9	132.4 ± 8.7	131.9 ± 10.6	0.87
AG (mmHg)	19.5 ± 9.9	20.7 ± 10.2	21.2 ± 7.9	0.85
SBP right (mmHg)	164.7 ± 20.5	158.0 ± 23.1	159.8 ± 17.4	0.42
SBP left (mmHg)	159.1 ± 19.1	155.9 ± 18.6	157.6 ± 14.1	0.87
DBP right (mmHg)	80.9 ± 9.7	79.8 ± 8.37	79.4 ± 10.5	0.94
DBP left (mmHg)	79.8 ± 8.8	81.5 ± 11.8	79.7 ± 9.32	0.67
ABPI right (mmHg)	1.0 ± 0.3	1.1 ± 0.2	1.1 ± 0.2	0.64
ABPI left (mmHg)	1.0 ± 0.2	1.1 ± 0.2	1.0 ± 0.3	0.82

All data are presented as the means ± SDs. *pMAP* Peripheral mean arterial pressure, *cSBP* Central systolic blood pressure, *cDBP* Central diastolic blood pressure, *cPP* Central pulse pressure, *cMAP* Central mean arterial pressure, *AIx* Augmentation index, *AI hr75* Augmentation index heartrate 75, *HR* Heartrate, *TR time* Transistor time, *AG* Augmentation. Pressure: *SBP* Systolic blood pressure, *DBP* Diastolic blood pressure, *ABPI* Ankle brachial pressure index.

**Table 4:** Pressure and arterial stiffness according to FBN1 genotype in females

	2/2 genotype	2/3 genotype	2/4 genotype	
	*n* = 82	*n* = 18	*n* = 33	*p*-value
pMAP (mmHg)	103.8 ± 14.1	109.6 ± 11.6	106.7 ± 14.1	0.39
cSBP (mmHg)	139.5 ± 18	137.1 ± 23.3	138.3 ± 15.2	0.36
cDBP (mmHg)	78.7 ± 11.1	81.1 ± 10.1	79.3 ± 12.4	0.29
cPP (mmHg)	68.7 ± 16.4	71.1 ± 19.8	67.1 ± 15.8	0.88
cMAP (mmHg)	106.8 ± 17.1	109.6 ± 11.6	106.7 ± 14.1	0.39
AIx (%)	35.03 ± 9.39	39.86 ± 8.93	35.80 ± 8.95	0.047
AI hr75 (%)	25.8 ± 6.6	28.3 ± 6.6	26.8 ± 6.6	0.72
HR (beat/min)	69.9 ± 12.4	62.6 ± 10.4	67.8 ± 11.3	0.048
TR time (s)	127.0 ± 9.1	126.0 ± 10.6	127.1 ± 8.2	0.49
AG (mmHg)	24.7 ± 10.3	29.1 ± 12.4	24.7 ± 9.9	0.18
SBP right (mmHg)	168.9 ± 23.4	178.3 ± 25.6	169.9 ± 23.4	0.14
SBP left (mmHg)	164.1 ± 19.6	174.6 ± 23.1	161.5 ± 17.4	0.049
DBP right (mmHg)	80.4 ± 11.32	80.7 ± 12.5	79.8 ± 10.1	0.94
DBP left (mmHg)	79.5 ± 10.8	80.0 ± 12.5	78.3 ± 11.4	0.84
ABPI right (mmHg)	1.0 ± 0.2	1.0 ± 0.1	1.0 ± 0.2	0.41
ABPI left (mmHg)	1.0 ± 0.2	1.1 ± 0.1	1.0 ± 1.2	0.48

All data are presented as the means ± SDs. *pMAP* Peripheral mean arterial pressure, *cSBP* Central systolic blood pressure, *cDBP* Central diastolic blood pressure, *cPP* Central pulse pressure, *cMAP* Central mean arterial pressure, *AIx* Augmentation index, *AI hr75* Augmentation index heartrate 75, *HR* Heartrate, *TR time* Transistor time, *AG* Augmentation. Pressure: *SBP* Systolic blood pressure, *DBP* Diastolic blood pressure, *ABPI* Ankle brachial pressure index

There was a significant difference in HR between the three main genotypes in the male group. The 2/2 genotype showed a significantly higher HR (*p* = 0.036) than the 2/3 and 2/4 genotypes. After adjustments for age, the statistically significant difference compared to the 2/3 and 2/4 genotypes disappeared.

In the female group, the 2/3 genotype showed a significantly higher AIx (*p* = 0.029) and SBP (*p* = 0.025) than the 2/2 and 2/4 genotypes. The difference in AIx between the genotypes was still significant after adjustment for age (*p* = 0.031). Regarding SBP, the 2/3 genotype showed a slight increase in SBP after adjustment for age; however, no significant difference between the 2/3 genotype and the 2/2 or 2/4 genotype was observed. Furthermore, the 2/3 genotype in females had a significantly lower HR (*p* = 0.048) than the 2/2 and 2/4 genotypes.

## Discussion

This study shows that elderly hypertensive females have a higher SBP, pMAP, cMAP, AIx, AG and TR than elderly hypertensive males. We have also shown a higher SBP and a higher AIx among hypertensive elderly females with the FBN1 2/3 genotype compared to hypertensive females with the two other common genotypes (2/2 and 2/4). Small but not statistical significantly differences in AIx between the FBN1 genotypes were also seen in male.

Increased arterial stiffness results in elevated systolic blood pressure, which is a prognostic indicator of future cardiovascular events [23, 24]. However, the Framingham study showed an increase in the incidence of cardiovascular diseases in females with age, and the cardiovascular incidence seems to be higher after menopause [25]. The lower level of estrogen in postmenopausal women could be the main reason for the increased risk of cardiovascular diseases [26, 27]. Many postmenopausal females are treated with hormone replacement therapy (HRT), and Mueck et al. found that both normotensive and hypertensive postmenopausal women run a very low risk of blood pressure increase during HRT [28].

Increases in SBP, cSBP, central and peripheral MAP, and cPP were observed in the hypertensive elderly females of our study compared to males, and these findings are in accordance with those of previous reports [29–31]. We used an oscillometric technique to measure brachial blood pressure, and a noninvasive tonometric system was used to analyze the pulse wave of the radial artery. Furthermore, when blood pressure was adjusted for age, HR and BSA, the differences between the sexes increased. Goto et al. (2013) used invasive measurements of blood pressure and observed the same trend in a large cohort, with a higher SBP and AIx (*p* = 0.048) among elderly females than among elderly males, similar to our findings (Table III) [31].

Elderly hypertensive females seem to have higher SBP than males; thus, it is important to discuss the differences between sexes regarding the treatment of hypertension. Ong et al. (2008) studied sex differences in blood pressure rate control among Americans with diagnosed hypertension and showed that blood pressure measurement control rates were not significantly lower among females than among males [32]. The guidelines recommend that hypertensive males and females should be treated with the same approach, and evidence shows that females show a benefit from antihypertensive treatment that is similar to that of males. The 2018 ESH/ESC Guidelines recommend the use of antihypertensive drugs by all patients diagnosed with grade 1 hypertension [33]. The use of antihypertension drugs may affect the arterial impedance. In the present study we found a significance difference between sexes in diuretic treatment. However, diuretics seem to have no beneficial effect on pulsatile hemodynamics and a neutral effect on AIx and PWV. Beta-Blockers, ACE-inhibitors and Calcium channel blockers have been seen to improves arterial stiffness, but there was no significance difference between sexes in treatment with these medications [34].

FBN1 is a glycoprotein that forms FBN1-rich microfibrils and functions as a skeleton for the deposition of tropoelastin, forming elastic fibers in the arterial wall. FBN1 might affect the maintenance of the integrity of the arterial wall of central arteries, and FBN1 is considered to be a candidate gene of arterial stiffness [13, 35]. The different FBN1 genotypes have been studied in several populations, and the frequencies of these genotypes in our present study are in accordance with what others have reported previously among healthy subjects [17, 18, 36], AAA-patients [37], patients with coronary artery diseases [16] and middle-aged subjects [22]. The increased prevalence of the FBN1 2/3 genotype in this population may explain the impact on the structure of the aortic wall, as shown by increased aortic stiffness.

The females in the present study with the FBN1 2/3 genotype had a higher AIx and SBP than those with the other common genotypes, namely, 2/2 and 2/4. These differences were not observed in males. One causative explanation of the observed sex difference could be the fact that hypertensive males with high levels of and/or multiple risk factors for cardiovascular diseases already have died in cardiovascular events due to the early onset of cardiovascular morbidity in males [3, 4]. Arterial stiffness is an independent predictor of cardiovascular morbidity and mortality, and AIx is an indirect measure of arterial stiffness [3, 4]. Several studies have found an association between the FBN1 2/3 genotype and arterial stiffness in males [15, 16]; however, we observed an association between higher AIx and FBN1 2/3 in only elderly hypertensive females and not in elderly hypertensive males. Furthermore, after adjustment for age, the increase in AIx was reduced, although it still reached significance. In studies of both sexes (healthy individuals), no associations between the FBN1 2/3 genotype and arterial stiffness were observed [18]. Compared to that of the previously published work, our population consisted of hypertensive elderly patients, and this could be a possible explanation for the difference in findings. Arterial aging is associated with a decreased amount of elastin in the large elastic arteries and affects arterial stiffness. As arterial stiffness is an independent predictor of cardiovascular morbidity and mortality, some elderly hypertensive patients with high levels of and/or multiple risk factors for cardiovascular diseases may have already died due to cardiovascular events. Thus, it must be considered that the differences between the genotypes may be larger in a younger population.

### Limitations

The interpretation of the findings from the present study has limitations. First, the different number of subjects in each FBN1 genotype group (Tables 3 and 4) could influence the results due to limited numbers of subjects in the FBN1 2/3 genotype group. Second, the PWA was calculated from measurements of the radial artery and was performed according to the manufacturer’s recommendations and guidelines regarding measurements of PWA [38]. However, peripheral blood pressure may not accurately reflect actual aortic PWV. Third, our analysis follows a cross-sectional design and no data on outcomes are reported; therefore, no direct cause-and-effect associations can be derived from this study design. A fourth limitation is that no lifestyle items, medical history of stroke and information about diabetic microvascular complications were included. Our participants were old and the duration of diabetes and HbA1c was not included in the study. Finally, it is a limitation that we do not have data about left ventricular ejection fraction and valvular heart disease. More studies are needed to define the possible effect that the FBN1 2/3 genotype may have on cardiovascular morbidity and mortality.

## Conclusion

In conclusion, the findings of our study indicate that hypertensive females in an elderly population have increased SBP and arterial stiffness compared to males. These findings also indicate that elderly females with FBN1 2/3 have increased arterial stiffness compared to hypertensive elderly females with FBN1 2/2 or FBN1 2/4. Therefore, these findings may be used to design potential therapeutic targets to reduce hypertension in females with the FBN1 2/3 genotype. Measurement of PWV may be a useful tool in clinical practice, especially in female, to select subjects at high risk of developing cardiovascular diseases [39]. However, further studies are needed to confirm our results.

## Data Availability

The datasets used and/or analyzed during the current study are available from the corresponding author on reasonable request.

## Abbreviations

AAA: Abdominal aortic aneurysm
ABPI: Ankle brachial pressure index
AugP: Augmentation pressure
AI hr75: Augmentation index heartrate 75
AIx: Augmentation index
AG: Augmentation pressure
BMI: Body mass index
BSA: Body surface area
BP: Base pair
cMAP: Central mean arterial pressure
cPP: Central pulse pressure
cSBP: Central systolic blood pressure
DBP: Diastolic blood pressure
ECG: Electrocardiographically
FBN1: Fibrillin-1
HDL: High-density lipoprotein
HR: Heart rate
HRT: Hormone replace therapy
LDL: Low-density lipoprotein
MFS: Marfan syndrome
MI: Myocardial infarction
PCR: Polymerase chain reaction
pMAP: Peripheral mean arterial pressure
PP: Pulse pressure
PWA: Pulse wave analysis
PWV: Pulse wave velocity
SBP: Systolic blood pressure
TR time: Transistor time
VNTR: Variable number of tandem repeat
